# Vitamin D receptor gene polymorphisms affecting changes in visceral fat, waist circumference and lipid profile in breast cancer survivors supplemented with vitamin D3

**DOI:** 10.1186/s12944-019-1100-x

**Published:** 2019-08-09

**Authors:** Elham Kazemian, Atieh Amouzegar, Mohammad Esmaeil Akbari, Nariman Moradi, Safoora Gharibzadeh, Yasaman Jamshidi-Naeini, Maryam Khademolmele, Atefeh As-habi, Sayed Hossein Davoodi

**Affiliations:** 1 Endocrine Research Center, Research Institute for Endocrine Sciences, Shahid Beheshti University of Medical Sciences, Tehran, Iran; 2 Department of Basic Sciences and Cellular and Molecular Nutrition, Faculty of Nutrition Sciences and Food Technology and National Nutrition and Food Technology Research Institute, Shahid Beheshti University of Medical Sciences, Tehran, Iran; 3 Cancer Research Center, Shahid Beheshti University of Medical Sciences, Tehran, Iran; 4 Department of Biochemistry, Faculty of Medicine, Kurdistan University of Medical Sciences, Sanandaj, Iran; 5 Department of Biochemistry, Faculty of Medicine, Iran University of Medical Sciences, Tehran, Iran; 6 Department of Epidemiology and Biostatistics, Research Centre for Emerging and Reemerging Infectious Disease, Pasteur Institute of Iran, Tehran, Iran; 7 Department of Nutritional Sciences, Texas Tech University, Lubbock, TX USA; 8 Department of Nutrition Science, Faculty of Medical Science and Technology, Science and Research Branch (SRBIAU), Islamic Azad University, Tehran, Iran; 9 Food Safety Research Center, Semnan University of Medical Sciences, Semnan, Iran; 10 Department of Nutrition, School of Nutrition and Food Sciences, Semnan University of Medical Sciences, Semnan, Iran; 11 National Nutrition and Food Technology Research Institute, Faculty of Nutrition Sciences and Food Technology, Shahid Beheshti University of Medical Sciences, No 7, west Arghavan st. Farahzadi Blv, Shahrake Gharb, Postal Code, Tehran, 1981619573 Iran

**Keywords:** Vitamin D3, Plasma 25(OH)D, Breast cancer survivor, Anthropometric measures, Lipid profile, Vitamin D receptor

## Abstract

**Objective:**

We investigated whether vitamin D receptor (*VDR*) polymorphisms are associated with circulating metabolic biomarkers and anthropometric measures changes in breast cancer survivors supplemented with vitamin D3.

**Methods:**

One hundred sixty-eight breast cancer survivors admitted to Shohaday-e-Tajrish hospital received 4000 IU of daily vitamin D3 supplements for 12 weeks. Anthropometric measurements as well dietary, physical activity and plasma metabolic biomarkers assessments were performed before and after intervention. *VDR* polymorphisms were considered as the main exposures. Multivariate multiple linear regression analyses were used to determine the association between the *VDR* single-nucleotide polymorphisms (SNPs) and changes in metabolic and anthropometric measures in response to vitamin D3 supplementation.

**Results:**

One hundred twenty-five (85%) women had insufficient and inadequate levels of plasma 25-hydroxy vitamin D (25(OH)D) at baseline. Compared to the AA genotype of the *ApaI*, the aa category showed greater increase in muscle mass [71.3(10.7131.9)] and higher decrease in LDL-C [− 17.9(− 33.6, − 2.3)] levels after adjustment for potential confounders. In addition, the heterozygous genotype (Bb) of the *BsmI VDR* was associated with higher increase in WC following vitamin D3 supplementation, compared to BB [2.7(0.1,5.3)]. Haplotype score analyses indicate a significant association between inferred haplotypes from *BsmI*, *ApaI*, *TaqI* and *FokI*, *BsmI* and *Cdx2 VDR* polymorphisms and on-study visceral fat changes.

**Conclusions:**

Findings of this study showed that genetic variation in the VDR gene was associated with changes in cardio-metabolic parameters in breast cancer survivors, supplemented with vitamin D3, results could provide a novel insight into better understanding of which subset of individuals benefit most from normalization of vitamin D status.

**Trial registration:**

This trial has been registered on the Iranian Registry of Clinical Trials (IRCT) under the identification code: IRCT2017091736244N1, registration date: 2017-11-10, http://www.irct.ir/trial/27153 and was approved by the ethics committees of the National Nutrition and Food Technology Research Institute (NNFTRI), Shahid Beheshti University of Medical Sciences (SBMU).

**Electronic supplementary material:**

The online version of this article (10.1186/s12944-019-1100-x) contains supplementary material, which is available to authorized users.

## Introduction

Obesity is a multifactorial metabolic disorder prevalent worldwide and a potential risk factor for many life threatening non communicable diseases, including cancer [1]. Many biological (genetics) and environmental determinants (diet) contribute to the pathogenesis of obesity, characterized by increase in adipocyte number and size [2].

Evidence from cellular, animal and epidemiological studies indicate that vitamin D plays a protective role in the onset of obesity via the inhibition of adipogenesis, inducing adipocyte apoptosis and enhanced fatty acid oxidation [3, 4]; furthermore, vitamin D promotes lipid mobilization and utilization in the adipocytes resulting in improved adipose tissue metabolic function [5, 6]. The vitamin D receptor (*VDR*), a key mediator of vitamin D pathway, is expressed in human pre- and differentiated adipocytes [3, 7]. The 1,25-dihydroxyvitamin D, the active metabolite of vitamin D, binds to the *VDR* and forms a heterodimer with the retinoid-X receptor (*RXR*) [7]; *RXR*-*VDR* heterodimers enter the nucleus and modulate the transcription of target genes, including adipogenic gene expression which contributes to regulation of weight gain and visceral adiposity [3, 8, 9].

Obesity is well known to be implicated in the development and recurrence of breast cancer [10]; here we hypothesize that vitamin D action in adipose tissue may partially explain the role of vitamin D in cancers. The most common *VDR* polymorphisms reported to be associated with cancer and obesity are *BsmI* (rs1544410), *ApaI*(rs7975232), *TaqI* (rs731236), *FokI* (rs2228570) and *Cdx2* (rs 11,568,820) [11, 12]. Likewise, previous epidemiologic studies indicate that obesity is associated with low levels of 25-hydroxy vitamin D3(25(OH) [13]. Despite the significant effects of vitamin D and its receptor on energy metabolism and anthropometric traits [3, 12], human studies assessing the role of vitamin D in obesity, document contradictory results. One explanation could be due to the genotypic effects that may be observed only in specific environmental conditions [14], e.g. example, evidence suggests that *VDR* polymorphisms may interact with circulating 25(OH)D levels and alter the risk of clinical outcomes [14]. It hence seems reasonable to hypothesize that genetic variations within the *VDR* gene could alter the individual’s response to vitamin D3 supplementation in terms of obesity and metabolic status. Demonstrating the interactions of the *VDR* and vitamin D intake by trials of vitamin D supplementation, would facilitate identification of subjects who have benefited most from vitamin D interventions. To best of our knowledge, this is one of the first studies to analyze whether the variation in the *VDR* gene (*BsmI*, *ApaI*, *TaqI*, *FokI* and *Cdx2*) could modulate the effects of vitamin D3 supplementation on anthropometric measures and metabolic biomarkers among breast cancer survivors.

## Material and methods

This analysis was part of a larger trial conducted on breast cancer survivors. Briefly, one hundred sixty-eight women with invasive or in situ carcinoma, who were admitted to Shohaday-e-Tajrish hospital in Tehran and had completed the treatment protocols, including surgery, radio- and chemotherapy, received 4000 IU of daily vitamin D3 supplement for 12 weeks. Health benefits of vitamin D, without increasing the risk of overdose, were demonstrated by plasma 25(OH)D levels of 75 to 110 nmol/l, obtained in the range of 1800 to 4000 IU vitamin D3 intakes per day [15]. it is noteworthy that although the study intervention was limited to the winter and spring months; there was no significant difference in circulating 25(OH)D levels during these seasons in Tehranian women [16]. The study was restricted to women, aged 25–65 years, body mass index (BMI) ≥ 25 kg/m2, no use of either vitamin D3 supplements ≥1000 IU/day for at least 4 months before entry to the study or dietary and herbal supplements during the intervention period. Exclusion criteria were history of malabsorption syndrome, calcium metabolism disorders, gastrointestinal, renal, inflammatory (sarcoidosis, etc.) and other endocrinological diseases, under treatment for weight reduction, high levels of 25(OH)D concentration (≥100 nmol/liter), use of medication interfering with vitamin D metabolism and absorption and pregnancy. Demographic, background and pathologic data were collected through face to face interviews and from medical records. Anthropometric measurements including BMI, waist circumference (WC), hip circumference (HC), body composition analyses as well dietary, physical activity and plasma metabolic biomarkers assessments were conducted before and after supplementation and for comparisons of outcomes between different *VDR* polymorphic groups.

Subjects were contacted by telephone every 2 weeks to assess their adherence to the supplementation regimen, which was determined by dividing the numbers of pills consumed by the numbers prescribed. Subjects not consuming more than 10% of tablets were excluded. All participants read and signed informed consent forms at the beginning of the study. This trial has been registered on the Iranian Registry of Clinical Trials (IRCT) under the identification code: IRCT2017091736244N1, registration date: 2017-11-10, http://www.irct.ir/trial/27153 and was approved by the ethics committees of the National Nutrition and Food Technology Research Institute (NNFTRI), Shahid Beheshti University of Medical Sciences (SBMU). All methods were performed in accordance with the relevant guidelines and regulations.

### Study measurements

#### Anthropometrics measurements

Height was measured to the nearest 0.1 cm using the Secastadiometer. Weight, WC and HC were measured at the beginning and end of the intervention. Weight was measured with light clothes, without shoes, using a digital scale to the nearest 0.1 kg. BMI was calculated by dividing weight (Kg) by height (m^2^). We measured WC (cm) over light clothing at midway between the lowest rib and the iliac crest and HC at largest circumference of the buttocks. Body fat percentage and distribution were assessed by bioelectrical impedance analysis (BIA) (Tanita BC-418, Illinois, USA). All assessment was performed by trained nutritionists.

#### Physical activity assessment

Physical activity measurement was done at the beginning and end of the study, using the International Physical Activity Questionnaire (IPAQ) translated by the Iran's National Elites Foundation and overall physical activity was reported as Metabolic Equivalent Task minutes per week (MET-min/wk) [17].

#### Sun exposure assessment

Sun exposure was determined at enrollment and at end of study by assessing hours/week spent an outdoor activity and body surface area (BSA) exposed to sunlight while outdoors. Then, a sun exposure index was calculated by multiplying the percentage of BSA exposed to sunlight by the hours of sun exposure per week [18].

#### Dietary intake assessment

Dietary intakes at the beginning and end of study were evaluated by 3-day food record including a weekend day and 2 working days.

#### Laboratory measurements

All laboratory assessments were carried out at the laboratory of the Department of biochemistry, Faculty of Medicine, Iran University of Medical Sciences. Fasting venous blood samples were taken from all participants at enrollment and end of intervention. DNA was extracted from WBC and DNA quality was determined using a Nanodrop spectrophotometer by measuring ratio of the absorbance at 260 and 280 nm. Polymerase chain reaction (PCR)- Restriction Fragment Length Polymorphism (RFLP) methods were employed for *VDR* genotyping at *FokI*, *ApaI*, *TaqI*, *BsmI* single-nucleotide polymorphisms (SNPs) and tetra arms PCR method for Cdx-2 [19].

Lipid profiles including plasma total cholesterol (TC), low-density lipoprotein cholesterol (LDL-C), high-density lipoprotein cholesterol (HDL-C) and triglycerides (TG) were measured using the enzymatic calorimetric method by the auto-analyzer (Roche Hitachi 912, Basel, Switzerland).

Plasma 25(OH)D concentrations were measured by ELISA kit (Euroimmun, Lübeck, Germany;intra- and inter-assay coefficients of variation (CVs) were 5 and 7.8% respectively).

#### Statistical analyses

We used the Shapiro-Wilk test to assess distribution of variables. The differences between two continuous variables with normal and skewed distribution were determined by the paired sample t-test and Wilcoxon test respectively. The *ApaI*, *TaqI*, *FokI*, *BsmI* and *Cdx2 VDR* polymorphisms were considered as the main exposures. Changes in variables during the study were calculated by subtracting the value of pre- from the post intervention and variables with skewed distribution were log-transformed. Regression analysis was conducted on data of 143 participants, who took ≥90% of their pills, with no gaps > 5 days and no other vitamin D supplementation during the study.

Because we have more than one outcome and the outcomes are clinically related, we used multivariate multiple linear regression analyses to avoid the problem of multiple testing; this would arise if the effects of each confounder on each dependent variable were tested separately. In addition, false-discovery rate (FDR) methods were employed to correct for multiple comparisons in haplotype analyses [20]. For the first regression, we considered metabolic factors as a vector of response and in the second regression the anthropometric measures were considered as vectors of response. Metabolic vectors included LDL-C, HDL-C, TC, TG while BMI, WC, HC, fat mass, and muscle mass, trunk and visceral fat were considered as anthropometric vectors. The factors known or hypothesized to be associated with obesity and plasma lipid profiles, including age, menopause status, medication including lipid and glucose lowering agent, on-study 25(OH)D changes as well plasma 25(OH)D levels, energy and fat intake and physical activity (as continues variables) at baseline were regarded as predictors. We considered *VDR* as main exposures one at a time. To avoid multicollinearity, we did not enter waist and hip with waist to hip ratio in the same model; to do this we entered vitamin D at baseline and changes in vitamin D separately, one at a time. In addition, to answer the question whether the effects of *VDR* gene polymorphisms (as a main exposure) on lipids and anthropometric measures could be pleiotropic (direct effect) or mediated via changes in plasma 25(OH)D levels (as an intermediate variable) on the causal pathway between the main exposure and the outcome of interest, causal mediation analysis was conducted [21].

Distributions from the Hardy–Weinberg equilibrium (HWE) for each SNP were examined using an exact test, performed by the HWE exact function in the R package “HardyWeinberg”. *P* value less than 0.05 was considered significant. R package ‘haplo.stats’ was used to test the association of estimated haplotypes with changes in response variables [22], haplo score was adjusted for age, 25(OH) D changes during the study, 25(OH)D level, energy intake and physical activity at baseline. All *P* values were presented for a two-tailed test and P values < 0.05 were considered statistically significant. Statistical analyses were performed using Stata14.0 (StataCorp. 2015). The computing environment R Version 3.4.3 (R Development Core Team, 2017) was used to perform statistical analysis.

## Results

The final analysis was conducted on the data of 147 overweight and obese subjects, previously diagnosed with breast cancer at Shohadaye Tajrish hospital; median survival time for the study participants since diagnosis was 3 years. Eighty-three (56%) women were diagnosed with ER^+^PR^+^ and 18(12%) with triple negative breast cancer respectively. Thirty-three (22%) patients had stage I, 61(41%) had stage II and 35(24%) had stage III at first diagnosis; median age of subjects was 50 years and 112(76%) women were post-menopausal. Eighty (54%) patients performed exercise at low intensity, while only 23(16%) of subjects had physical activity of vigorous intensity. Participants’ average energy, carbohydrate, protein and fat intakes over the course of the day, at baseline were 1901 Kcal, 243, 56 and 84 g respectively (Table 1). Baseline circulating 25(OH)D levels ranged from 2.4 to 180.2 nmol/liter (median,38.1; interquartile range (IQR), 22.9–62.6) and reached a median of 108.8 (IQR, 73.2–142.0) at the end of the study period after vitamin D treatment. One hundred twenty-five (85%) women had deficient and insufficient levels of plasma 25(OH)D at baseline, while 109 (74%) participants were found to be vitamin D sufficient at the end of the intervention period, based on the definition of 25(OH)D level < 50, 50–75 and ≥ 75 nmol/liter as deficient [23], insufficient and sufficient respectively [24]. After the 12-week supplementation, the mean plasma 25(OH)D change was 65.3 nmol/liter. All genotypes distributions were in Hardy–Weinberg equilibrium proportions.

**Table 1:** Basic characteristics of the study participants

Characteristics	Total
Age	50(43–55)
Current smoking, N (%)	1(1)
Hormone receptor status, N (%)	
ER+	89(60)
ER + PR+	83(56)
ER + PR-	5(3)
ER-PR-	35(23)
HER2+	12(8)
ER-PR-HER2-	18(12)
Family history of breast cancer, N (%)	25(17)
Post-menopausal, N (%)	111(76)
Hormone therapy for breast cancer	112(76)
Stage, N (%)	
Stage I	33(22)
Stage II	61(41)
Stage III	35(24)
Missing	18(13)
Recurrence N (%)	
Yes	5(3)
No	77(67)
Unknown	65(30)
Physical activity levels, N (%)	
Low physical activity	80(54)
Moderate physical activity	44(30)
High physical activity	23(16)
Energy intake, Kcal	1901(1832–2046)
Fat intake, grams	84 ± 16
Protein intake, grams	56(49–62)
Carbohydrate intake, grams	243 ± 41
Pre intervention Vitamin D, nmol/liter	38(22–62)
Post intervention vitamin D, nmol/liter	110 ± 46
*ApaI* N (%)	
AA	68(49)
Aa	52(38)
aa	18(13)
*TaqI N (%)*	
TT	57(41)
Tt	67(49)
tt	14(10)
*BsmI N (%)*	
BB	66(45)
Bb	65(44)
bb	16(11)
*FokI* N (%)	
FF	64(44)
Ff	66(45)
ff	17(12)
*Cdx2* N (%)	
GG	81(55)
AG	52(35)
AA	14(10)

*ER* Estrogen Receptor, *PR* Progesterone Receptor, *HER2* Human Epidermal Growth Factor Receptor 2, *BMI* Body Mass Index, *BMI* Body Mass Index, *WC* Waist Circumference, *HC* Hip Circumference

Values with normal distribution are presented as mean ± SD, values with non-normal distribution as median (Q1, Q3) and categorical variables as N (%)

We found significant changes in HC and trunk fat within individuals before and after treatment with vitamin D3: HC (median pre-treatment, 109.5 vs median post-treatment, 107.5) and trunk fat (median pre-treatment, 33.0 vs median post-treatment, 34.0). No significant changes were observed in BMI, WC, fat mass, muscle mass, visceral fat, LDL-C, HDL-C, TC and TG within individuals before and after vitamin D3 supplementation (Table 2).

**Table 2:** Comparison of changes in response variables before and after vitamin D3 supplementation (4000 IU/day) for 12 weeks

Variables	Before intervention (*n* = 147)	After intervention (*n* = 147)	P value*
25(OH)D, nmol/liter	38.1(22.9–62.6)	108.8(73.2–142.0)	< 0.001
BMI, Kg/m2	29.4(27.3–31.9)	29.2(27.3–32.1)	0.57
WC, cm	96.0(91.0–103.7)	98.5(92.2–104.0)	0.20
HC, cm	109.5(105.2–114.0)	107.5(104.0–112.5)	< 0.001
Fat mass (%)	37.15 ± 4.16	37.4 ± 4.14	0.08**
Muscle mass (%)	43.7(41.3–47.3)	43.7(41.0–46.7)	0.42
Trunk fat (%)	33.0(29.3–36.8)	34.0(30.4–37.4)	0.003
Visceral fat level	8.0(7.0–10.0)	8(7.0–10.0)	0.42
LDL, mg/dL	101.8 ± 26.1	98.7 ± 26.5	0.19
HDL, mg/dL	52.0(45.0–59.0)	51(46.0–60.0)	0.86
TC, mg/dL	176.0(157.0–193.5)	167.0(153.5–195.5)	0.17
TG, mg/dL	108.0(80.5–145.0)	108.0(77.5–148.0)	0.92

Values with normal distribution are presented as mean ± SD; values with non-normal distribution are presented as median (Q1, Q3)

*P values were calculated using Wilcoxon signed-rank test

**P values were calculated using the paired sample t-test Wilcoxon test respectively

*25(OH)D* 25-hydroxy vitamin D, *BMI* Body mass index, *WC* Waist circumference, *HC* Hip circumference, *TC* Total cholesterol, *LDL-C* Low-density lipoprotein cholesterol, *HDL-C* High-density lipoprotein cholesterol, *TG* Triglycerides

Estimated differences in plasma metabolic biomarkers and anthropometric measures in response to vitamin D3 supplementation per variant allele are depicted in Table 3. The *ApaI VDR* polymorphisms was associated with plasma LDL-C levels and muscle mass changes in breast cancer survivors supplemented with vitamin D3. Compared to the AA genotype of *Apa1*, the aa group was accompanied with larger increase and decrease in muscle mass [71.3(10.7131.9)] and LDL-C [− 17.9(− 33.6, − 2.3)] after adjustment for age, energy and fat intake at baseline, baseline physical activity and plasma 25(OH)D levels respectively. In addition, the heterozygous genotype (Bb) of the *BsmI VDR* variant was associated with higher increase in WC in response to intervention compared to BB [2.7(0.1,5.3)]. Likewise, compared to the TT group, individuals with Tt genotypes of the *TaqI VDR* had a marginally significant increase in HC after vitamin D3 supplementation [1.9(− 0.1,3.9)]. The Cdx2 genotype AA was associated with larger decrement in plasma LDL-C levels, compared to GG [− 18.1(− 35.3, − 0.9)]. No significant association was detected between the *Cdx2 VDR* and anthropometric measures.

**Table 3:** Associations of VDR SNP genotypes and metabolic and anthropometric measures in response to vitamin D3 supplementation (4000 IU/day) in breast cancer survivors using multivariate multiple regression

Changes in varibles	*APa1*, β (95% CI)		*TaqI*, β (95% CI)		*BsmI*, β (95% CI)		*FokI*, β (95% CI)		*Cdx2*, β (95% CI)	
	Aa (*n* = 52)	aa (*n* = 18)	Tt (*n* = 67)	tt (*n* = 14)	Bb (*n* = 65)	bb (*n* = 16)	Ff (*n* = 66)	ff (*n* = 17)	GA (*n* = 52)	AA (*n* = 14)
BMI, Kg/m^2^ *P* value	0.13 (−0.17,0,44) 0.39	0.21 (− 0.23, 0.66) 0.35	− 0.06 (− 0.37,.24) 0.66	0.03 (− 0.45,0.53) 0.87	0.08 (− 0.21,0.38) 0.58	0.06 (− 0.41,0.54) 0.78	0.01 (− 0.27,0.31) 0.90	− 0.2 (− 0.6,0.2) 0.37	−0.16 (− 0.46. 0.14) 0.29	0 (− 0.48,0.50) 0.96
WC, cm *P* value	−0.4 (−3.3, 2.3) 0.72	0.8 (−3.2,4.8) 0.69	−1.4 (−4.1,1.3) 0.31	− 1.3 (−5.7,3.1) 0.55	2.7 (0.1,5.3) 0.04	3.9 (− 0.2,8.1) 0.06	1.2 (− 1.3,3.9) 0.34	−0.8 (− 4.9,3.2) 0.68	1.8 (− 0.8,4.6) 0.17	−1.2 (− 5.6, 3.2) 0.59
HC, cm *P* value	0.9 (− 1.1,3.0) 0.37	0.9 (−2.0,4.0) 0.53	1.9 (− 0.1,3.9) 0.06	0.6 (− 2.6,3.9) 0.70	0.9 (− 1.0,2.9) 0.32	0.1 (− 3.0, 3.3) 0.93	1.4 (− 0.5,3.3) 0.15	0.1 (− 2.9, 3.1) 0.93	−0.8 (− 2.9,1.1) 0.41	0.17 (− 3.1,3.5) 0.91
Fat mass (%) *P* value	0.08 (− 0.70,0.86) 0.83	0.53 (− 0.59,1.66) 0.34	−0.1 (− 0.9,0.6) 0.70	−0.2 (− 1.5,0.9) 0.66	0.2 (− 0.5, 1.0) 0.53	0.5 (− 0.6,1.8) 0.3	0.4 (− 0.3,1.1) 0.28	−0.8 (− 2.0,0.3) 0.14	0.06 (− 0.74,0.87) 0.86	−0.2 (− 1.5, 1.1) 0.75
Muscle mass (%) *P* value	0.2 (−41.9,42.3) 0.99	71.3 (10.7131.9) 0.02	− 22.0 (− 63.9,19.8) 0.30	− 25.7 (− 93.3,41.8) 0.45	− 22.4 (− 62.1,17.3) 0.26	− 26.7 (− 90.6,37.2) 0.41	19.7 (− 19.9,59.4) 0.32	1.7 (− 59.7,63.2) 0.95	20.3 (− 20.6,61.3) 0.32	− 6.8 (− 73.8,60.1) 0.84
Trunk fat (%) *P* value	0. 2 (− 0.8,1.3) 0.67	0.5 (− 1.0,2.1) 0.65	− 0.06 (− 1.15, 1.02) 0.90	−0.1 (− 1.9, 1.5) 0.84	0.3 (− 0.7,1.4) 0.58	0.7 (− 0.9,2.5) 0.38	0.5 (− 0.5,1.6) 0.34	− 1.0 (− 2.7,0.6) 0.22	0.2 (− 0.9,1.3) 0.71	− 0.2 (− 2.1,1.5) 0.77
Visceral fat *P* value	0.1 (− 0.2,0.4) 0.5	0.2 (− 0.2,0.7) 0.37	− 0.1 (− 0.4,0.2)	−0.1 (− 0.7,0.3)	− 0.04 (− 0.37,0.27) 0.77	0.3 (− 0.1,0 .8) 0.17	0.1 (− 0.1,0.4) 0.32	−0.2 (− 0.7,0.2) 0.38	−0.03 (− 0.3,0.3) 0.82	−0.04 (− 0.6,0.5) 0.86
LDL, mg/dL *P* value	− 4.0 (− 15.0,6.8) 0.46	− 17.9 (− 33.6, − 2.3) 0.02	0.6 (− 10.1,11.3) 0.90	7.4 (− 10.0,24.9) 0.40	3.9 (− 6.3,14.2) 0.44	2.1 (− 14.5,18.8) 0.79	3.2 (− 7.0,13.5) 0.53	−2.5 (− 18.5,13.5) 0.75	−6.5 (− 16.9,3.8) 0.21	− 18.1 (− 35.3, − 0.9) 0.03
HDL, mg/dL *P* value	0.9 (− 3.5,5.4) 0.68	2.3 (− 4.0,8.8) 0.46	−2.2 (− 6.5,2.06) 0.30	0.84 (− 6.1,7.8) 0.81	− 1.8 (− 5.9,2.3) 0.38	3.3 (− 3.2,10.0) 0.31	1.4 (−2.6.5.5) 0.48	− 0.6 (− 7.10,5.7) 0.83	− 0.7 (− 5.0, 3.4) 0.72	1.2 (− 5.7, 8.2) 0.72
TC, mg/dL *P* value	−4.1 (− 18.9,10.7) 0.58	−4.8 (− 26.0,16.3) 0.65	−9.7 (− 23.9,4.4) 0.17	3.7 (− 19.3,26.7) 0.75	3.8 (− 10.1,17.9) 0.58	11.4 (− 11.3,34.2) 0.32	−1.2 (− 15.3,12.7) 0.85	−3.8 (− 25.7,18.0) 0.72	−9.8 (− 24.1, 4.4) 0.17	−22.1 (− 45.7, 1.3) 0.06
TG, mg/dL *P* value	6.3 (− 14.1,26.8) 0.54	− 13.1 (− 42.4,16.2) 0.37	2.5 (− 17.3,22.4) 0.79	21.2 (− 11.0,53.6) 0.19	8.6 (− 10.4, 27.7) 0.37	−4.5 (− 35.4, 26.3) 0.77	−9.4 (− 28.4,9.5) 0.32	6.2 (− 23.3,35.8) 0.67	− 3.0 (− 22.6,16.4) 0.75	−25.9 (− 58.1, 6.2) 0.11

*BMI* Body mass index, *WC* Waist circumference, *HC* Hip circumference, *TC* Total cholesterol, *LDL-C* Low-density lipoprotein cholesterol, *HDL-C* High-density lipoprotein cholesterol, *TG* Triglycerides

All values were adjusted for age, baseline 25-hydroxy vitamin D (25(OH)D), energy intake, fat intake and physical activity

Haplo.score analyses were carried out considering plasma metabolic biomarkers and anthropometric measure changes as a quantitative trait (Table 4 and Additional file 1: Table S1). At first, we evaluated a combination of the set of all SNPs determined in the current study and then, based on findings of other studies [19–21], 3-SNP haplotype markers was constructed to determine the effect of haplotype blocks on changes in plasma metabolic biomarkers and anthropometric measure in response to vitamin D3 supplementation. Haplotype blocks were constructed as follows: H1: *Cdx2*, *FokI*, *BsmI*, *ApaI*, *TaqI*; H2: *Cdx2*, *FokI*, *BsmI*, H3: *BsmI*, *ApaI*, *TaqI* and H4, *FokI*, *BsmI*, *ApaI*. Haplotype score analyses indicated that HC (global score statistic = 26.97, *p* value = 0.04), visceral fat (global score statistic = 45.46, p value< 0.001) and HDL-C (global score statistic = 27.83, p value = 0.03) changes were associated by H1. Moreover, a significant association of changes in circulating HDL-C (global score statistic = 16.43, p value = 0.02) and TC (global score statistic = 15.02, p value = 0.03) with the H1 haplotype and significant relation of changes in visceral fat (global score statistic = 79.69, p value< 0.001) and plasma TG (global score statistic = 13.48, p value = 0.03) with H3 and H4 haplotypes was identified respectively (Table 4). However, only the association of visceral fat with H1 and H3 haplotypes remained significant after FDR correction. We found the GFbAT (Haplo.Score = − 2.27, p value = 0.02) and GFbAt (Haplo.Score = − 2.04, p value = 0.04) haplotypes were negatively associated with changes in visceral adiposity after vitamin D3 treatment.

**Table 4:** Haplotype block analysis of metabolic and anthropometric changes after vitamin D supplementation (4000 IU/day) for 12 weeks

Variables	H1			H2			H3			H4		
	Global-Stat	*P*_Value_*	FDR P _Value_**	Global-Stat	*P*_Value_*	FDR P _Value_**	Global-Stat	*P*_Value_*	FDR P _Value_**	Global-Stat	*P*_Value_*	FDR *P*_Value_**
BMI, Kg/m2	18.87	0.27	0.65	3.89	0.79	0.92	9.94	0.19	0.65	5.61	0.46	0.72
WC, cm	10.66	0.82	0.92	5.47	0.60	0.86	8.05	0.32	0.65	6.73	0.34	0.65
HC, cm	26.97	0.04	0.25	4.68	0.69	0.92	7.33	0.39	0.66	2.88	0.82	0.92
Fat mass, cm	17.33	0.63	0.86	7.66	0.36	0.65	3.96	0.78	0.92	8.26	0.21	0.65
Muscle mass, (%)	13.50	0.85	0.92	4.28	0.74	0.92	7.60	0.36	0.65	11.36	0.07	0.34
Trunk fat, (%)	17.50	0.62	0.86	9.01	0.25	0.65	2.64	0.91	0.93	7.13	0.30	0.65
Visceral fat	45.46	< 0.001	< 0.001	3.58	0.82	0.92	79.69	< 0.001	< 0.001	7.82	0.25	0.65
LDL, mg/dL	18.90	0.27	0.65	7.53	0.37	0.65	9.37	0.22	0.65	9.20	0.16	0.64
HDL, mg/dL	27.83	0.03	0.22	16.43	0.02	0.22	5.74	0.56	0.84	9.24	0.16	0.64
TC, mg/dL	15.83	0.46	0.72	15.02	0.03	0.22	2.90	0.89	0.93	6.41	0.37	0.65
TG, mg/dL	−53.73	1	1.00	3.24	0.86	0.92	13.06	0.07	0.34	13.48	0.03	0.22

*BMI* Body mass index, *WC* Waist circumference, *HC* Hip circumference, *TC* Total cholesterol, *LDL-C* Low-density lipoprotein cholesterol, *HDL-C* High-density lipoprotein cholesterol, *TG* Triglycerides

H1, *Cdx2*, *FokI*, *BsmI*, *ApaI*, *TaqI*; H2, *Cdx2*, *FokI*, *BsmI*, H3, *BsmI*, *ApaI*, *TaqI*; H4, *FokI*, *BsmI*, *ApaI*

*All values were adjusted for age, baseline 25-hydroxy vitamin D (25(OH)D), energy intake, fat intake and physical activity

**FDR P values were obtained using the false-discovery rate (FDR) methods

As depicted in Table 5, the results of mediation analyses indicated that the effects of the *VDR* genetic variation on lipids and WC were not mediated via its effect on plasma 25(OH) D level (indirect).

**Table 5:** Total, direct, and indirect effects of *VDR* SNPs on LDL-C, WC and muscle mass percentage for plasma 25-hydroxy vitamin D (25(OH)D changes as the mediator

Outcomes	Exposures	ACME (95% CI)^a^		ADE (95% CI)^b^		Total effect (95% CI)		Proportion mediated 95% CI	
LDL-C (mg/dL)	*ApaI*	aa	Aa	aa	Aa	aa	Aa	aa	Aa
		−0.07 (−3.58, 3.45)	0.01 (−0.99, 1.26)	−17.92 (−34.00, −2.29)	−4.17 (−15.00, 6.35)	− 17.99 (− 34.12, −2.21)	−4.15 (− 15.10, 6.52)	0.004 (− 0.33, 0.32)	0.008 (− 0.65, 0.77)
	*Cdx2*	GG	GA	GG	GA	GG	GA	GG	GA
		−0.15 (−3.22, 2.94)	− 0.01 (−1.06, 0.87)	−17.41 (− 32.73, − 0.60)	−6.12 (− 16.10, 3.56)	−17.57 (− 32.93, − 0.73)	−6.14 (− 16.23, 3.57)	0.007 (−0.25, 0.31)	0.001 (− 0.50, 0.35)
WC (cm)	*BsmI*	bb	Bb	bb	Bb	bb	Bb	bb	Bb
		0.01 (−0.02, 0.06)	−0.002 (− 0.02, 0.02)	0.16 (− 0.09, 0.44)	0.08 (− 0.08, 0.25)	0.17 (− 0.08, 0.46)	0.07 (− 0.09, 0.26)	0.02 (− 0.90, 0.91)	−0.003 (− 0.94, 0.85)
Muscle mass (%)	*ApaI*	aa	Aa	aa	Aa	aa	Aa	aa	Aa
		−0.43 (−3.28, 1.66)	−0.38 (−2.21, 0.80)	−8.40 (− 23.36, 7.26)	−12.73 (− 21.86, − 3.31)	−8.83 (− 23.96, 6.75)	−13.12 (− 22.49, − 3.44)	0.01 (− 1.15, 0.93)	0.01 (−0.09, 0.19)

^a^average causal mediation effects, ^b^average direct effects

Adjusted for age, baseline Body Mass Index (BMI), energy intake, fat intake and physical activity

*LDL-C* Low-density lipoprotein cholesterol, *WC* Waist circumference

AA, GG and BB were considered as reference category for *ApaI*, *Cdx2* and *BsmI* respectively

## Discussion

We found that changes in WC and circulating LDL-C were associated with the *VDR ApaI* SNPs whereas changes in muscle mass were associated with the *BsmI* SNPs in breast cancer survivors supplemented with vitamin D3. Moreover, visceral fat changes were associated by the inferred haplotypes from the *ApaI*, *TaqI*, *FokI*, *BsmI* and *Cdx2* SNPs following 12-weeks of vitamin D3 supplementation.

Body fat mass [25–28], trunk fat [26, 29], WC, waist to hip ratio (WHR) [26, 28, 30] and serum lipids [22, 31] have been reported to be negatively associated with the changes in circulating 25(OH)D levels following vitamin D supplementation in some, but not all studies [30, 32–36]. In addition, studies investigating the association of genetic polymorphisms and haplotypes of *VDR* with adiposity and metabolic measures have shown equivocal results [8, 12, 37–43]. Nevertheless, without considering both *VDR* genotypes, vitamin D intake and circulating 25(OH) D data simultaneously, we could not determine the complex interplay between genetic variation in the *VDR* gen, and vitamin D with obesity or metabolic syndrome. There is limited data presenting the associations of vitamin D intake or circulating 25(OH)D with obesity and metabolic biomarkers, based on common genetic differences in the *VDR*. For example, Levin et al. in a discovery cohort of 1514 old white participants noted that the association between circulating 25(OH)D and health outcomes including hip fracture, myocardial infarction (MI) and cancer could be modulated by the common genetic variation in the *VDR* [44]. However, it should be kept in mind that their analyses were limited to a single measurement of serum 25(OH)D at baseline which may not be a good representative for average levels of circulating 25(OH)D during different times. Vimaleswaran et al. examined the effects of interaction between the rs7968585 and rs223917 *VDR* polymorphisms and circulating 25(OH)D on cardio-metabolic outcomes, including BMI, WC, WHR and lipid profiles in the 1958 British Birth cohort; no significant interactions were found except the interaction between rs2239179 SNP and circulating 25(OH)D on WHR [43]. In contrast with our findings, in a randomized control trial of 60 type 2 diabetic patients, results showed that vitamin D supplementation (25 μg/d) decreased obesity indices, including WC, body fat mass and truncal fat, only in individuals with the AA genotype of *Cdx2 VDR* [26]. Such contradictory results may be due to differences in the subjects, especially gender and menopause-associated changes which could reduce activation of vitamin D and the expression of *VDR* protein [45, 46]. In a study conducted among postmenopausal women, individuals having the *TaqI* t allele in combination with the *FokI* f alleles were more responsive to calcitriol therapy in terms of preventing recurrent vertebral fracture [47].

The *VDR* polymorphisms studied, showed no effects on BMI and fat mass in response to vitamin D3 supplementation in our analyses, similar to the findings of Zittermann et al. who showed that although WC was significantly decreased in response to vitamin D3 treatment, body weight and fat mass were not altered [32].

Further, haplotype analyses suggested that the specific combinations of alleles inferred from the *ApaI*, *TaqI*, *FokI*, *BsmI* and *Cdx2* were associated with visceral adiposity changes in breast cancer survivors treated with vitamin D3. To be more specific, we found a significant association of GFbAT and GFbAt haplotypes with visceral fat changes after vitamin D3 supplementation. Few previous studies have examined *VDR* SNP haplotypes as predictors of obesity and metabolic outcomes; however, their results may not be fully comparable with our findings due to the different *VDR* SNPs haplotypes investigated in these studies. Beydoun et al. reported a positive relation between the BAt haplotype of the *BsmI*, *ApaI*, and *TaqI* polymorphisms of the *VDR* and metabolic syndrome [48]. In addition, a negative association of bAT haplotypes with significant increase in central adiposity was indicated among African American adults [49]. Likewise, in another study conducted by Al-Daghri et al. the *VDR TaqI* (G), *BsmI* (T) *ApaI* (A) haplotype was significantly associated with the risk of obesity [7]. Our findings highlight the utility of considering the combined effects of several variants of *VDR* gene on vitamin D related health outcomes. Nevertheless, our study lacked enough power to precisely estimate this association and future studies in diverse and larger samples are needed to verify our results.

From a biological point of view, the active form of 1,25-dihydroxyvitamin D (1,25(OH)2D) contributes to obesity and lipid metabolism in various ways including: 1) increased adipocytes apoptosis 2) enhanced fatty acid oxidation, 3) upregulation of uncoupling protein expression, and 4) reduced lipolysis [47, 48]. The 1,25(OH)2D binds to *VDR* to form the vitamin D-*VDR* axis that interacts with DNA to promote or suppress vitamin D target genes [7, 47]. It has been shown that the *BsmI*, *ApaI*, and *TaqI* SNPs, positioned within the 3′ untranslated region of the *VDR* gene, affects either *VDR* mRNA stability or *VDR* transcriptional activity [50] whereas *FokI* SNP, located in the translational initiation site of *VDR*, results in generation of altered *VDR* protein activity [50] and *Cdx2* modifies the transcriptional activity of the *VDR* promoter region [51]. Therefore, any changes in this axis, including low levels of 25(OH)D or genetic variation in the *VDR* gene could alter energy, adipocyte and lipid metabolism, e.g. increased *VDR* expression in adipocytes reduces energy expenditure resulting in increased adipose mass [7]. Additionally, longer *VDR BsmI* and polyA repeats represent lower mRNA stability and decreased translation of the *VDR* protein, resulting in in reduced vitamin D response such as inhibiting of adipocytes and muscle mass differentiation [52].

To summarize, this is the first work studying the association of the *VDR* SNPs and haplotypes with metabolic status and obesity indices in response to vitamin D3 supplementation among breast cancer survivors. Our study has several strengths including its controlled trial design, adjustment for potential covariates interfering with outcomes of interest, high prevalence of vitamin D deficiency among participants and haplotype analysis. However, the current research has its limitations which need to be addressed. First, the most important limitation of the current study is its small sample size resulting in inadequate power and hence the negative findings in our analyses. Second, the 12-week trial may not be long enough to see the possible effects of vitamin D on adiposity measures and lipid metabolism. Third, we did not measure body composition by Dual X-Ray absorptiometry (DXA) which is considered as the gold standard. Nevertheless, the BIA methods is regarded as a reliable and validated method to measure body composition. Lastly, but not least, we did not investigate the other candidate genes involved in vitamin D metabolism e.g. *RXR* nuclear receptor and genes contributing to obesity such as *UCP1*, *UCP2*, *ADRA2B*, *ADRB3*, *LEPR*, and *ESR1* and their interaction with the *VDR*; these analyses may contribute to a better understanding of environmental and gene interactions. Future functional studies of *VDR* gene, well-designed clinical trials with larger sample size and longer intervention periods and comprehensive assessment of other candidate gene involved in obesity and vitamin D metabolism may validate our findings.

In conclusion, the *VDR* gene variations can modify the effects of vitamin D3 supplementation on WC, HC, visceral fat and lipid profiles changes in breast cancer survivors, findings which provide a novel insight into a better understanding of which subsets of individuals are at greater risk of the adverse effects of vitamin D deficiency or those who benefit most from normalization of vitamin D status.

## Additional file

Additional file 1: **Table S1.** Haplo. Score analysis of visceral fat changes after vitamin D supplementation (4000 IU/day) for 12 weeks. (DOCX 17 kb)

## Data Availability

The datasets used during the current study are available from the corresponding author on reasonable request.

## Abbreviations

25(OH): 25-hydroxy vitamin D_3_
BIA: Bioelectrical impedance analysis
BMI: Body mass index
BSA: Body surface area
CVs: Coefficients of variation
DXA: X-Ray absorptiometry
FDR: False-discovery rate
HC: Hip circumference
HDL-C: High-density lipoprotein cholesterol
HWE: Hardy–Weinberg equilibrium
IPAQ: International physical activity questionnaire
IQR: Interquartile range
IRCT: Iranian registry of clinical trials
LDL-C: Low-density lipoprotein cholesterol
MI: Myocardial infarction
NNFTRI: National nutrition and food technology research institute
PCR: Polymerase chain reaction
RFLP: Restriction fragment length polymorphism
*RXR*: Retinoid-X receptor
SBMU: Shahid Beheshti university of medical sciences
SNPs: Single-nucleotide polymorphisms
TC: Total cholesterol
TG: Triglycerides
*VDR*: Vitamin D receptor
WC: Waist circumference
WHR: Waist to hip ratio
