# Correction to: Vitamin D receptor gene polymorphisms affecting changes in visceral fat, waist circumference and lipid profile in breast cancer survivors supplemented with vitamin D3

**DOI:** 10.1186/s12944-019-1110-8

**Published:** 2019-09-14

**Authors:** Elham Kazemian, Atieh Amouzegar, Mohammad Esmaeil Akbari, Nariman Moradi, Safoora Gharibzadeh, Yasaman Jamshidi-Naeini, Maryam Khademolmele, Atefeh As’habi, Sayed Hossein Davoodi

**Affiliations:** 1 Endocrine Research Center, Research Institute for Endocrine Sciences, Shahid Beheshti University of Medical Sciences, Tehran, Iran; 2 Department of Basic Sciences and Cellular and Molecular Nutrition, Faculty of Nutrition Sciences and Food Technology and National Nutrition and Food Technology Research Institute, Shahid Beheshti University of Medical Sciences, Tehran, Iran; 3 Cancer Research Center, Shahid Beheshti University of Medical Sciences, Tehran, Iran; 4 Department of Biochemistry, Faculty of Medicine, Kurdistan University of Medical Sciences, Sanandaj, Iran; 5 Department of Biochemistry, Faculty of Medicine, Iran University of Medical Sciences, Tehran, Iran; 6 Department of Epidemiology and Biostatistics, Research Centre for Emerging and Reemerging Infectious Disease, Pasteur Institute of Iran, Tehran, Iran; 7 Department of Nutritional Sciences, Texas Tech University, Lubbock, Texas USA; 8 Department of Nutrition Science, Faculty of Medical Science and Technology, Islamic Azad University, Science and Research Branch (SRBIAU), Tehran, Iran; 9 Food Safety Research Center, Semnan University of Medical Sciences, Semnan, Iran; 10 Department of Nutrition, School of Nutrition and Food Sciences, Semnan University of Medical Sciences, Semnan, Iran

**Correction to: Lipids Health Dis 2019 18:161**

**https://doi.org/10.1186/s12944-019-1100-x**

Following publication of the original article [1], the authors reported that one of the co-authors has a mistake in the author name. The correct spelling of author Atefeh As-habi is Atefeh As’habi. The original article [1] has been updated.
