# Supplementary material for: Vitamin D receptor gene polymorphisms affecting changes in visceral fat, waist circumference and lipid profile in breast cancer survivors supplemented with vitamin D3
Source: Lipids Health Dis. 2019 Aug 9;18:161. doi: 10.1186/s12944-019-1100-x (PMC6688337; doi:10.1186/s12944-019-1100-x)
Supplement: Supplementary file 1 — Table S1. Haplo. Score analysis of visceral fat changes after vitamin D supplementation (4000 IU/day) for 12 weeks. (DOCX 17 kb) [file 12944_2019_1100_MOESM1_ESM.docx]

**Additional file 1: Table S1**. Haplo. Score analysis of visceral fat changes after vitamin D supplementation (4000 IU/day) for 12 weeks

| **Haplotypes** | | | | | | **Hap-Freq** | **Hap-Score** | **P value** |
| --- | --- | --- | --- | --- | --- | --- | --- | --- |
| ***Cdx-2* *FokI* *BsmI* *ApaI* *TaqI*** | *Cdx2* | *FokI* | *BsmI* | *ApaI* | *TaqI* |  |  | **<0.001** |
| 1 | G | F | b | A | T | 0.05 | -2.27 | 0.02 |
| 2 | G | F | b | A | t | 0.04 | -2.04 | 0.04 |
| 3 | A | f | B | a | t | 0.02 | -1.65 | 0.09 |
| 4 | A | f | B | a | T | 0.02 | -1.29 | 0.19 |
| 5 | G | F | B | A | T | 0.13 | -0.98 | 0.32 |
| 6 | G | F | B | A | t | 0.15 | -0.70 | 0.48 |
| 7 | A | F | B | A | T | 0.04 | -0.58 | 0.55 |
| 8 | G | f | B | A | T | 0.03 | -0.24 | 0.80 |
| 9 | G | f | b | a | t | 0.02 | -0.00 | 0.99 |
| 10 | A | F | b | a | T | 0.02 | 0.19 | 0.84 |
| 11 | A | f | B | A | T | 0.02 | 0.29 | 0.76 |
| 12 | A | f | b | A | T | 0.02 | 0.30 | 0.76 |
| 13 | A | F | B | a | T | 0.05 | 0.34 | 0.72 |
| 14 | G | F | B | a | T | 0.07 | 0.55 | 0.58 |
| 15 | G | f | B | a | T | 0.04 | 0.66 | 0.50 |
| 16 | A | F | b | A | T | 0.02 | 0.84 | 0.40 |
| 17 | A | f | B | A | t | 0.01 | 1.10 | 0.26 |
| 18 | G | f | b | A | T | 0.06 | 1.24 | 0.21 |
| 19 | G | f | B | A | t | 0.02 | 1.30 | 0.19 |
| 20 | G | F | b | a | T | 0.04 | 1.77 | 0.07 |
| ***BsmI* *ApaI* *TaqI*** | | | | | | | | **<0.001** |
| 1 |  |  | b | A | t | 0.09 | -1.72 | 0.08 |
| 2 |  |  | B | A | T | 0.24 | -0.96 | 0.33 |
| 3 |  |  | B | a | t | 0.04 | -0.16 | 0.87 |
| 4 |  |  | B | A | t | 0.18 | 0.26 | 0.79 |
| 5 |  |  | B | a | T | 0.19 | 0.36 | 0.71 |
| 6 |  |  | b | A | T | 0.14 | 0.56 | 0.57 |
| 7 |  |  | b | a | T | 0.06 | 1.60 | 0.10 |

All values were adjusted for age, baseline 25-hydroxy vitamin D (25(OH)D), energy intake, fat intake and physical activity.

Hap-Freq is indicates the estimated frequency of the haplotype in the pool of all subjects.
